# NETs in the spotlight: exploring NETosis markers for tracking disease activity in IgA vasculitis

**DOI:** 10.1093/rheumatology/keaf272

**Published:** 2025-05-21

**Authors:** Vafa Guliyeva, Fatma Gül Demirkan, Erdem Bektaş, Rabia Deniz, Zeliha Emrence, Özlem Akgün, Selen Duygu Arık, Ayşenur Doğru, Ayşe Tanatar, Neslihan Abacı, Sema Sırma Ekmekci, Ahmet Gül, Nuray Aktay Ayaz

**Affiliations:** Department of Pediatric Rheumatology, Istanbul University Istanbul Faculty of Medicine, Istanbul, Turkey; Department of Pediatric Rheumatology, Istanbul University Istanbul Faculty of Medicine, Istanbul, Turkey; Department of Internal Medicine, Istanbul Faculty of Medicine, Istanbul University, Istanbul, Turkey; Department of Rheumatology, Başakşehir Çam and Sakura City Hospital, Istanbul, Turkey; Department of Genetics, Istanbul University, Aziz Sancar Institute of Experimental Medicine, Istanbul, Turkey; Department of Pediatric Rheumatology, Istanbul University Istanbul Faculty of Medicine, Istanbul, Turkey; Department of Pediatric Rheumatology, Istanbul University Istanbul Faculty of Medicine, Istanbul, Turkey; Department of Pediatric Rheumatology, Istanbul University Istanbul Faculty of Medicine, Istanbul, Turkey; Department of Pediatric Rheumatology, Istanbul University Istanbul Faculty of Medicine, Istanbul, Turkey; Department of Genetics, Istanbul University, Aziz Sancar Institute of Experimental Medicine, Istanbul, Turkey; Department of Genetics, Istanbul University, Aziz Sancar Institute of Experimental Medicine, Istanbul, Turkey; Division of Rheumatology, Department of Internal Medicine, Istanbul Faculty of Medicine, Istanbul University, Istanbul, Turkey; Department of Pediatric Rheumatology, Istanbul University Istanbul Faculty of Medicine, Istanbul, Turkey

**Keywords:** biomarkers, immunoglobulin A vasculitis, neutrophil extracellular traps (NETosis)

## Abstract

**Objectives:**

The role of neutrophil extracellular traps (NETs) in IgA vasculitis (IgAV) pathogenesis is emerging, with NETosis-associated markers potentially linked to disease activity. This study aimed to explore the relationship between NETosis biomarkers and IgAV disease phases.

**Methods:**

A longitudinal study involving 33 paediatric IgAV patients and 26 healthy controls was conducted. Blood and urine samples were collected from healthy controls and patients during active and inactive disease phases. NETosis markers, including cell-free DNA (cf-DNA), neutrophil elastase, MPO and citrullinated histone H3 (cit-H3) were measured using ELISA kits. Statistical analyses were conducted to compare differences for NETosis markers between groups and to evaluate correlations among variables using appropriate statistical tests.

**Results:**

There was no significant difference in gender and age between the patient and control groups. The serum cf-DNA level was significantly higher in the active patient group compared with the control and inactive patient groups (*P* = 0.04; *P* = 0.04, respectively). In urine, MPO levels were significantly lower in the active phase of patients than controls (*P* = 0.009), while cit-H3 levels were higher in both active and inactive phases compared with controls (*P* = 0.01 and *P* = 0.03, respectively). A cf-DNA threshold of 935 ng/ml was identified, which achieved a sensitivity of 93% (correctly identifying 93% of active patients) and a specificity of 72% (correctly identifying 72% of healthy controls).

**Conclusion:**

Elevated serum cf-DNA and urine cit-H3 suggest a potential role for NETosis in IgAV activity, highlighting these markers as potential indicators for disease monitoring. Further studies are warranted to establish standardized protocols for NETosis marker assessment in IgAV.

## Graphical abstract

**Figure:**
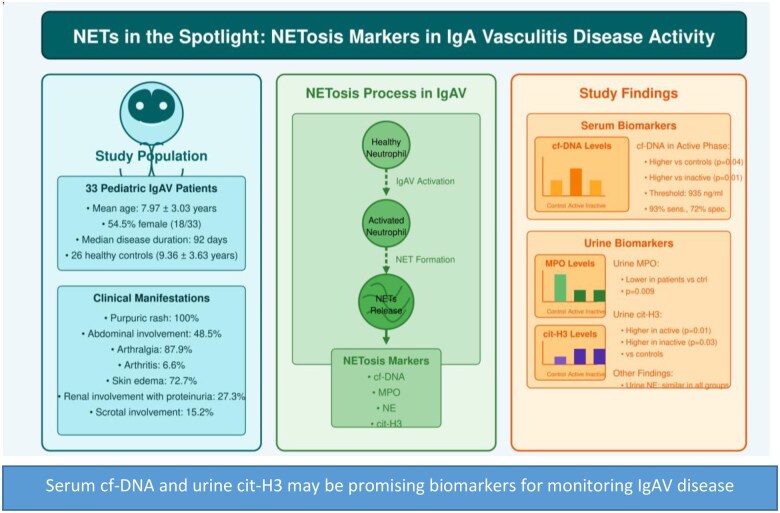

Rheumatology key messages

- Neutrophil extracellular traps (NETs) contribute to autoimmune diseases, but studies on paediatric IgA vasculitis (IgAV) research remains scarse.
- Serum cf-DNA levels increased during active IgAV, suggesting potential activity biomarker.
- Longitudinal monitoring of NETosis markers can aid in diagnosing and tracking IgA vasculitis phases.

## Introduction

IgA vasculitis (IgAV), the most common form of vasculitis in children, features non-thrombocytopenic purpura, gastrointestinal involvement and glomerulonephritis, with an annual incidence of 20–70 cases per 100 000 children [1, 2].

Its aetiology remains unclear, but is believed to involve an abnormal immune response to infections, drugs, vaccinations or environmental factors in genetically predisposed children. IgA accumulation in blood vessel walls suggests an IgA-mediated aberrant immune reaction to antigens [3]. Previous studies found that galactose-deficient IgA1 contributes to immune complex formation and their deposition in tissues including skin, gastrointestinal tract, joints and kidneys [3, 4].

Neutrophils contribute to disease pathogenesis through previously poorly understood mechanisms. A key discovery was neutrophil extracellular traps (NETs). ‘NETosis’ refers to cell death following neutrophil nuclear membrane disintegration, granule degranulation and DNA fragment release [5–7]. This discovery transformed our understanding the role of innate immunity in tissue damage, host defence, inflammation, thrombosis, autoimmunity and cancer [8, 9]. Targeting NETs shows promise for treating NET-associated diseases, highlighting the clinical relevance and therapeutic potential of this immunity aspect.

NETs have been linked to autoimmune and autoinflammatory conditions including SLE [10, 11], ANCA-associated vasculitis (AAV) [12], Behçet’s disease (BD) [13] and RA [14]. However, these studies have focused on adults. One solitary study has specifically investigated NETosis biomarkers in children with IgAV by examining the presence and amount of NETs in serum and tissue samples. However, as declared by authors due to the small number of biopsies (15 kidney biopsies and 9 gastrointestinal biopsies in patients with IgAV), the data may be subject to bias, and analysis of NETs in urine was not performed [15].

Despite being the most common childhood systemic vasculitis and typically self-limiting, understanding IgAV pathogenesis remains important due to potential gastrointestinal and renal complications. With unclear aetiopathogenesis, investigating NETosis in vascular inflammation is valuable. NETs may damage endothelial cells through cytotoxic components and serve as scaffolds for immune complex deposition, potentially representing a crucial disease mechanism. This investigation aims to elucidate the relationship between NETosis and vascular injury in IgAV, potentially identifying novel biomarkers while advancing our understanding of its pathophysiology.

We aimed to elucidate the role of NETosis in the pathogenesis of IgAV, and to explore the correlation between NETosis-associated biomarkers and disease activity. We hypothesize that NETosis-associated biomarkers could be valuable for both diagnosing and monitoring.

## Methods

### Study design and participants

This longitudinal experimental study was conducted between August 2023 and January 2024 in the Department of Pediatric Rheumatology, Istanbul University, Istanbul Faculty of Medicine. It included 33 IgAV patients under 18 years old and 26 age- and sex-matched healthy controls (HC). All patients met the clinical diagnosis of IgAV according to EULAR/PRINTO/PReS criteria [16]. Exclusion criteria included comorbidities affecting neutrophil function (like infections or malignancies) and prior treatment initiation. A total of 18 cases were omitted from the study (5 cases with comorbidities, 7 cases who had initiated treatment prior to presentation and 6 cases with incomplete data).

The control cohort consisted of hospital-based healthy volunteers and children undergoing routine health maintenance examinations, matched demographically with patients. The HC group had no documented medical conditions and was not taking any medications.

Blood and urine samples were collected from patients during both the active and inactive phases of the disease, as well as from controls.

Active phase was defined as the period before treatment initiation, marked by the onset of symptoms related to IgAV. None of the patients had received any treatment at the time of first sample collection. A second set of samples was obtained after patients entered the inactive phase (absence of purpura, abdominal pain or other IgAV symptoms).

Follow-up records included information on demographic data, recent infection history (within the past month), organ involvement, treatments, hospitalization history and laboratory test results (complete blood counts, CRP levels, ESR, complete urinalysis and protein/creatinine ratio in spot urine samples).

### Definition of clinical manifestations

- Fever: axillary temperature ≥37.8°C at presentation, or reported by parents within 48 h before presentation [17].
- Scrotal involvement: acute scrotal oedema with pain/tenderness on examination [18].
- Hypertension: systolic blood pressure (SBP) or diastolic blood pressure (DBP) ≥90th percentile, or SBP ≥120 mmHg or DBP ≥80 mmHg [19].
- Renal involvement: presence of haematuria and/or proteinuria [20].
- Abdominal/scrotal US and skin biopsy were performed when clinically indicated.

### Sample collection

Venous blood samples were collected into vacuum dry tubes, centrifuged at 3000 rpm for 10 min, and the obtained serum samples were aliquoted into cryotubes, stored at –80°C under appropriate conditions until further analysis.

Midstream urine samples of 5–10 ml were collected and gathered in 15 ml Falcon tubes, centrifuged at 3500 rpm for 3 min. The supernatants were aliquoted and stored in cryotubes at –80°C until analysis.

### Measurement of serum and urine cf-DNA NE, MPO and cit-H3 levels

Serum and urine cf-DNA levels were measured using Invitrogen Quant-iT PicoGreen dsDNA Assay Kit with a Qubit 2.0 fluorometer (ThermoFisher Scientific, Waltham, MA, USA). Serum and urine neutrophil elastase (NE), MPO and citrullinated histone H3 (cit-H3) levels were measured using commercial 96-well ELISA kits (for NE, Elabscience, E-ELH1946, Houston, TX, USA; for MPO, Hycult Biotech, HK324, Uden, The Netherlands; and for cit-H3, MyBiosource, MBS3804611, San Diego, CA, USA) were used according to the instructions and collected serum and urine samples were thawed once and used. Absorbance was measured at 450 nm wavelength on a microplate reader, and marker levels were calculated using standard values and expressed in ng/ml.

### Statistical analysis and ethics

Statistical analysis used SPSS version 22.0 for Windows (SPSS 22.0, Armonk, NY, USA). The Shapiro–Wilk test assessed data normality. Normally distributed data were presented as means ± s.d., non-normally distributed as medians with interquartile ranges (IQR, 25th–75th percentile) and categorical variables as percentages.

χ^2^ tests were used for categorical variables. For continuous variables, Student’s *t*-test (normal) or Mann–Whitney U test (non-normal) were used to compare independent groups, while paired *t*-test or Wilcoxon signed-rank test were used to compare paired samples. Correlations were assessed using Pearson’s (normal) or Spearman’s tests (non-normal). Statistical significance was *P* < 0.05.

Receiver operating characteristic (ROC) curve analysis evaluated the diagnostic value of biomarkers, assessing serum cf-DNA, urine cit-H3 and MPO levels’ ability to distinguish between active disease and controls. It also evaluated the ability of serum cf-DNA to differentiate active from inactive disease. GraphPad Prism V.10 was used for graphs. Power analysis with Gpower determined appropriate sample sizes, calculating 42 participants per group for 95% statistical power based on limited vasculitis NETosis studies.

The study was approved by Istanbul University Istanbul Faculty of Medicine Ethics Committee (2022-1460481), adhered to the Helsinki Declaration and obtained parental informed consent. Funding was provided by Istanbul University Scientific Research Projects Coordination Unit (project number: TSA-2023-39657).

## Results

### Clinical characteristics of the participants

The patient group consisted of 33 children, with 15 (45.5%) males and a mean disease onset age of 7.97 ± 3.03 years. The HC group included 26 individuals, with 11 (42.3%) males and a mean age of 9.36 ± 3.63 years. Gender and age were similar between patient and the control group. Table 1 summarizes the initial clinical characteristics of the patient cohort.

**Table 1.** Clinical characteristics of the patients

Characteristic	
No. of patients	33
Female (*n*, %)	18 (54.5)
Mean age ± s.d. (years)	7.97 ± 3.03
Median disease duration (days) (IQR 25–75)	92 (41–164)
History of viral infection within the last month (*n*, %)	15 (45.5)
Purpuric rash (*n*, %)	100 (100)
Median duration of purpuric rash (days) (IQR 25–75)	17 (13–30)
Abdominal involvement (*n*, %)	
Pain	16 (48.5)
Vomiting	7 (21.2)
Diarrhoea	2 (6.1)
Arthralgia (*n*, %)	29 (87.9)
Arthritis (*n*, %)	20 (6.6)
Myalgia (*n*, %)	23 (69.7)
Skin oedema (*n*, %)	
Hand–foot	24 (72.7)
Scalp	1 (3)
Fever (*n*, %)	4 (12.1)
Scrotal involvement (*n*, %)	5 (15.2)
Hypertension (*n*, %)	5 (15.2)
Laboratory parameters (mean ± s.d.)	
Hgb (g/dl)	12.2 ± 1.3
Platelet (/mm^3^)	407 400 ± 109 777
WBC (/mm^3^)	14 132 ± 5485
CRP (mg/l)	42.3 ± 36.9
ESR (mm/h)	17 ± 21
Diagnostic tests at the disease onset (*n*, %)	
Abdominal US	12 (36.3)
Scrotal US	3 (9)
Skin biopsy	2 (6)
Hospitalization	
Median duration (days) (IQR 25–75)	4.5 (3.2–6)
Indications (*n*, %)	
GIS involvement	12 (75)
Urogenital involvement	3 (18.7)
Musculoskeletal involvement	1 (6.3)
Treatment (*n*, %)	26 (78.8)
Median duration of steroid use (days) (IQR 25–75)	26 (14.5–45)
Pulse steroid therapy (*n*, %)	4 (12.1)
Steroid maintenance therapy (*n*, %)	14 (42.4)
MMF (*n*, %)	2 (6.1)

IQR: interquartile range; GIS: gastrointestinal; WBC: white blood cell.

At diagnosis, nine patients presented with proteinuria, having spot urine protein/creatinine ratios above 0.2 (median 0.27, IQR 0.22–0.57). During remission, proteinuria resolved in all except two cases (median spot urine protein/creatinine 0.15, IQR 0.13–0.19, *P* = 0.09). No patient was diagnosed with nephritis.

In the study cohort, 4 patients received pulse methylprednisolone (10–30 mg/kg/day for three consecutive days), while 14 patients underwent maintenance steroid therapy (1–2 mg/kg/day), typically tapered after approximately 1 week as disease severity decreased. The median duration of steroid therapy was 26 days (IQR 14.5–45).

### Comparative analysis of serum cf-DNA, MPO, cit-H3 and NE levels in patients with IgAV in active and inactive phases compared with HC

**Figure 1.**
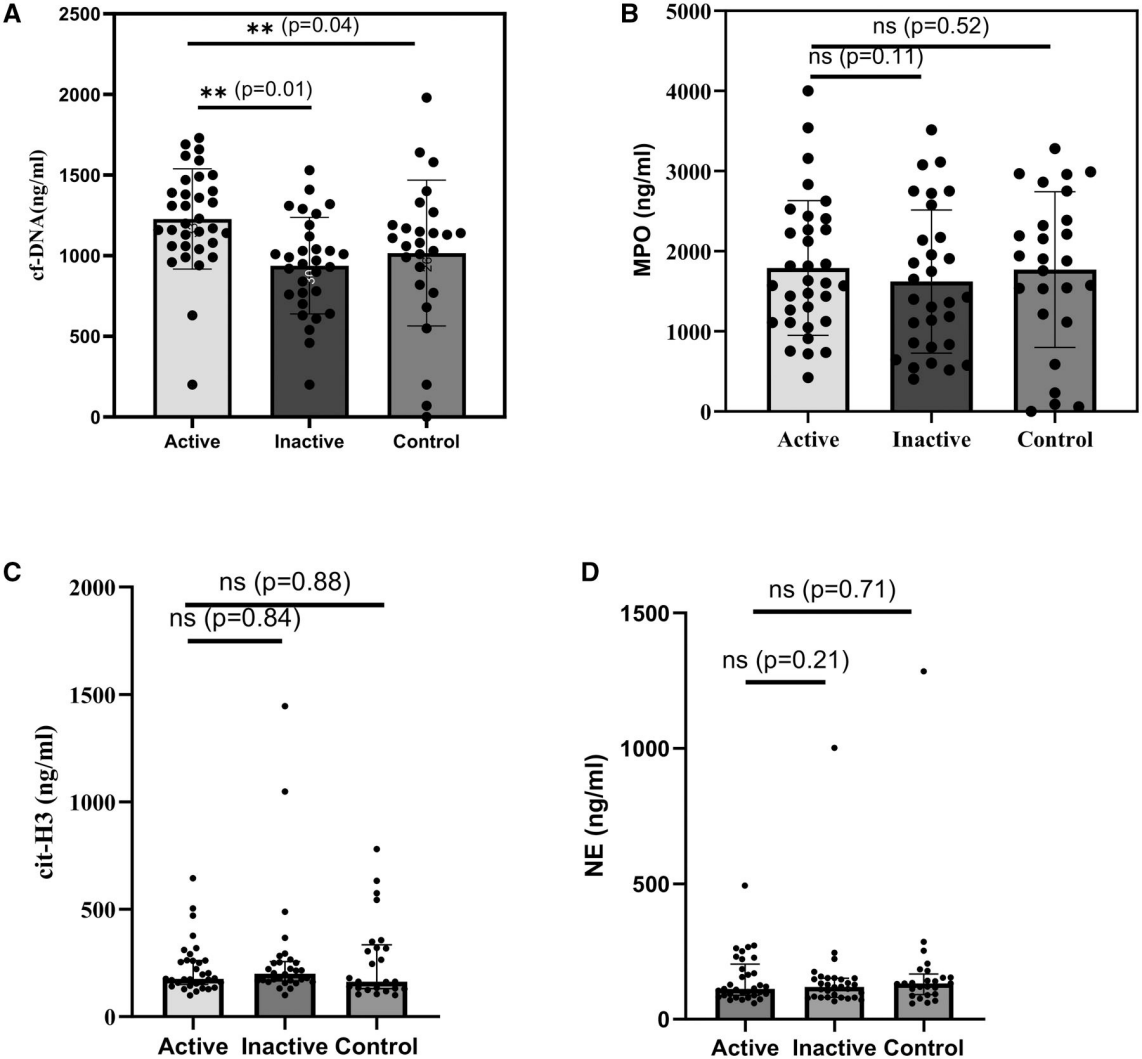
Levels of serum NETosis biomarkers in the control group, active and inactive patient groups. (**A**) Serum cf-DNA levels in the control group and IgAV group. (**B**) Serum MPO levels in the control group and IgAV group. (**C**) Serum cit-H3 levels in the control group and IgAV group. (**D**) Serum NE levels in the control group and IgAV group. Data are presented as median. Mann–Whitney U test; **P ≤ 0.05, ns: not significant. NET: neutrophil extracellular trap; IgAV: IgA vasculitis; cf-DNA: cell-free DNA; NE: neutrophil elastase; cit-H3: citrullinated histone-3

The control group had a median cf-DNA level of 1110 ng/ml (IQR 875–1230). Patients in the active phase showed significantly higher serum cf-DNA levels than controls (median 1200 ng/ml, IQR 1060–1435) (*P* = 0.04). In the inactive phase, patient serum cf-DNA levels (median 960 ng/ml, IQR 745–1137.5) were comparable to controls (*P* = 0.13) and significantly lower during the inactive phase than in the active phase (*P* = 0.01) (Fig. 1).

Median serum MPO values were higher in the control group (1904.26 ng/ml) than in patients during active (1602.18 ng/ml) and inactive (1412.53 ng/ml) phases, but no significant difference was observed. Similarly, median serum cit-H3 and NE levels in controls were comparable to those in patients during both active and inactive disease phases. No significant changes in MPO, cit-H3 or NE levels were observed between active and inactive phases in IgAV patients (Table 2).

**Table 2.** Comparison of urine and serum levels of MPO, cit-H3 and NE in patients with IgAV during active and inactive phases and in healthy controls

	Median MPO (ng/ml) (IQR 25–75)		Median cit-H3 (ng/ml) (IQR 25–75)		Median NE (ng/ml) (IQR 25–75)	
	Serum	Urine	Serum	Urine	Serum	Urine
Patient in active phase	1602.18 (1117.05–2338.43)	283.28 (234.03–904.4)	176.49 (148.74–261.15)	145.69 (136.24–196.75)	112.33 (88.18–203.12)	673.27 (416.77–1069.48)
Patient in inactive phase	1412.53 (828.93–2272.63)	280.88 (182.23–948.96)	199.28 (166.67–256.07)	159.7 (136.75–193.85)	118.82 (80.71–151.24)	650.51 (445.28–922.27)
Control	1904.26 (1372.55–2568.86)	1279.88 (310.11–1716.1)	163.36 (130.21–334.19)	141.88 (125.21–155.91)	132.16 (90.8–166.45)	725.1 (431.87–950.99)
*P*	a–i: 0.11	a–i: 0.78	a–i: 0.84	a–i: 0.64	a–i: 0.21	a–i: 0.31
	i–c: 0.25	i–c: 0.04*	i–c: 0.49	i–c: 0.01*	i–c: 0.41	i–c: 0.51
	a–c: 0.52	a–c: 0.009*	a–c: 0.88	a–c: 0.03*	a–c: 0.71	a–c: 0.77

* *P* < 0.05 statistically significant. IQR: interquartile range; a: active phase; i: inactive phase; c: control; NE: neutrophil elastase; cit-H3: citrullinated histone-3; IgAV: IgA vasculitis.

### Comparative analysis of urine cf-DNA, MPO, cit-H3 and NE levels in patients with IgAV in active and inactive phases compared with HC

Urine cf-DNA levels were comparable between the control group (median 92 ng/ml, IQR 49.5–165) and patients in the active phase (median 62 ng/ml, IQR 35–150) with *P* = 0.31 and *P* = 0.21, respectively. In the longitudinal evaluation, patients’ urine cf-DNA levels during the inactive phase (median 72.5 ng/ml, IQR 35.75–130) remained comparable to their levels during the active phase.

Urine median levels of MPO was significantly lower both in the active and the inactive phases (280.88 and 283.28 ng/ml, respectively) of the patients compared with the control group (1279.88 ng/ml) (*P* = 0.009 and *P* = 0.04, respectively). Median cit-H3 levels in urine was significantly higher in active (145.69 ng/ml) and inactive phases (159.7 ng/ml) compared with controls (141.88 ng/ml) (*P* = 0.01 and *P* = 0.03, respectively). Urine NE levels were comparable between patient and control groups. Table 2 provides a summary of serum and urine analyses across disease phases compared with controls.

No statistically significant differences in serum or urine NETosis biomarker levels were found between patients with and without abdominal or scrotal involvement. Similarly, no differences in serum or urine NETosis markers were observed between patients with and without kidney involvement in either active or inactive phases compared with controls.

### Correlation analysis of serum and urine NETosis biomarkers among groups

When examining correlations between serum biomarkers across patient phases and controls, a moderate negative correlation was found between serum cit-H3 and serum NE in the whole cohort (r = –0.536, *P* = 0.006**). In both active and inactive patient phases, no correlations were found among serum biomarkers.

For urine biomarkers, a positive correlation was observed between urine cf-DNA and urine NE, moderately evident in both controls and the active patient phase (r = 0.581/0.457, *P* = 0.006/0.007**).

A positive correlation was noted between serum MPO and urine MPO levels in both active and inactive disease phases. Serum cit-H3 and urinary cf-DNA levels were negatively correlated in the control group and active phase. Additionally, significant negative correlations between serum cit-H3 and urine MPO and significant positive correlation between serum cit-H3 and urine cit-H3 were found in both active and inactive patient phases (Table 3).

**Table 3.** Correlation analysis between serum and urine NETosis biomarkers in patients and control groups

		Active (*n* = 33)		Inactive (*n* = 33)		Control (*n* = 26)	
Serum	Urine	r	*P*	r	*P*	r	*P*
cf-DNA	cf-DNA	0.043	0.812	0.025	0.895	–0.233	0.323
	MPO	–0.193	0.282	–0.197	0.297	–0.101	0.672
	cit-H3	–0.089	0.624	0.002	0.993	–0.023	0.925
	NE	–0.055	0.760	–0.036	0.850	–0.008	0.975
MPO	cf-DNA	–0.066	0.716	–0.147	0.438	–0.063	0.791
	MPO	*0.403* *	*0.020* *	*0.432* *	*0.017* *	0.032	0.895
	cit-H3	–0.209	0.244	–0.001	0.995	–0.111	0.640
	NE	0.052	0.773	–0.344	0.063	–0.053	0.826
cit-H3	cf-DNA	*–0.345*	*0.049*	0.006	0.975	*–0.455* *	*0.044* *
	MPO	–0.042	0.815	*–0.375* *	*0.041* *	–0.153	0.519
	cit-H3	*0.440* *	*0.010* *	–0.265	0.156	0.017	0.945
	NE	–0.331	0.060	0.077	0.687	–0.176	0.458
NE	cf-DNA	0.045	0.802	0.080	0.673	0.255	0.278
	MPO	–0.139	0.441	–0.080	0.675	–0.039	0.870
	cit-H3	0.014	0.937	0.022	0.906	–0.104	0.663
	NE	0.222	0.215	0.007	0.971	0.137	0.565

(*) indicates a *P*-value ≤ 0.05, based on Spearman correlation analysis. NET: neutrophil extracellular trap; cf-DNA: extracellular free DNA; NE: neutrophil elastase; cit-H3: citrullinated histone-3.

### ROC analysis

The biomarkers showing significant differences between the control group and patients' active disease periods were evaluated using ROC curves. Serum cf-DNA levels and urine cit-H3 and MPO levels were analysed in both the active patient phase and the control group Serum cf-DNA was specifically evaluated to distinguish the inactivation phase of the disease.

The area under the curve (AUC) value for serum cf-DNA was 0.654 (95% CI 0.509–0.798, *P* = 0.046), with a threshold value of 935 ng/ml, demonstrating 93% sensitivity and 72% specificity to identify active disease. For urine cit-H3, the AUC value was 0.671 (95% CI 0.528–0.814, *P* = 0.035), with a threshold of 132.62 ng/ml, showing 85% sensitivity and 39% specificity. The AUC value for urine MPO was 0.711 (95% CI 0.550–0.873, *P* = 0.009) with a threshold value was 1096.96 ng/ml, exhibiting 81% sensitivity and 57% specificity to identify active disease.

When these threshold values were analysed using χ^2^ analysis for providing additional validation in the active phase of the patient group *vs* the control group, statistically significant differences were observed (serum cf-DNA, *P* = 0.03; urine cit-H3, *P* = 0.05; and urine MPO, *P* = 0.003).

As a result of validation, it was shown that serum cf-DNA and urine cit-H3 values higher than the threshold values and urine MPO values lower than the threshold values distinguished active patients from controls.

**Figure 2.**
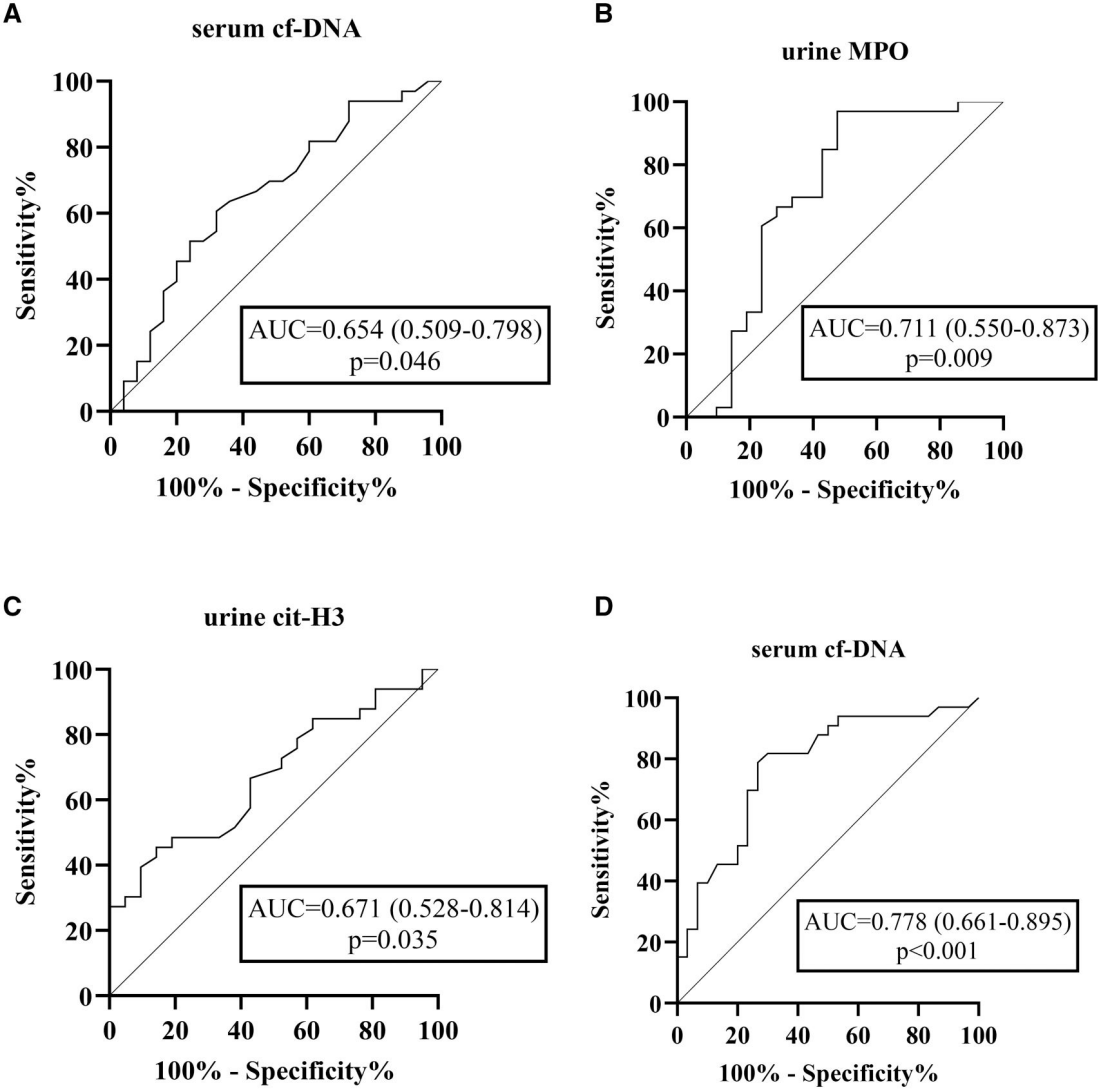
ROC curves of NETosis biomarkers for distinguishing active IgAV patients from controls and from those in the inactive disease phase. (**A**) ROC curve of serum cf-DNA comparing the active patient group and healthy controls. (**B**) ROC curve of urinary MPO comparing the active patient group and healthy controls. (**C**) ROC curve of urinary cit-H3 comparing the active patient group and healthy controls. (**D**) ROC curve of serum cf-DNA comparing the active and inactive phases in the patient group. ROC: receiver operating characteristic; NET: neutrophil extracellular trap; cit-H3: citrullinated histone-3; cf-DNA: cell-free DNA

ROC analysis was performed to assess serum cf-DNA’s ability to identify disease inactivation. The AUC was 0.778 (95% CI 0.661–0.895, *P* < 0.001), showing good diagnostic accuracy. A cf-DNA threshold of 1035 ng/ml was established, providing 82% sensitivity (correctly identifying 82% of inactive cases) and 70% specificity (correctly identifying 70% of active cases). χ^2^ testing confirmed this threshold’s statistical significance (*P* < 0.001) for distinguishing inactive from active phase patients (Fig. 2).

## Discussion

NETosis involves neutrophil cell death following activation, and imbalances in this process can cause persistent inflammation and tissue damage, potentially contributing to various inflammatory diseases [21]. NETs were first linked to vasculitis pathogenesis in AAV in 2009, with elevated NET levels found in circulation and kidney tissue [12]. Based on this intriguing information, we simultaneously assessed NETosis markers in blood and urine samples in children with IgAV. In comparison with controls, serum cf-DNA levels were found significantly elevated during the active phase of the disease. This variance may drop a hint to the role of NETs in disease activity in children with IgAV.

In a pioneering study, Bergqvist and colleagues detected NETs in various immune-complex (IC) -mediated cutaneous small vessel vasculitis by direct immunofluorescence staining. Skin biopsy of 21/72 adult patients with IgAV were positive for NETs, particularly in areas of dense vascular infiltration. NET density strongly correlated with vascular inflammation severity and reactive oxygen species production, especially in IgAV and hypersensitivity vasculitis [22]. These findings support our hypothesis that neutrophil activation and NET formation may play a crucial role in IgAV pathogenesis. Our longitudinal results extend Bergqvist’s observations by showing NET formation peaks at disease onset and declines during remission, suggesting NETs contribute to initial vascular damage rather than serving only as a consequence of inflammation. This temporal relationship between NETs and disease activity points to potential therapeutic targets in neutrophil activation pathways. While Bergqvist’s cross-sectional study examined only skin biopsies, our comprehensive approach examining NETosis markers in both serum and urine samples provides a more complete picture of neutrophil activity throughout the disease course, enhancing understanding of the systemic nature of this vasculitis.

Literature shows varied results for NETosis markers. Takeuchi *et al.* (2022) investigated the correlation between serum MPO-DNA and IgA levels in 22 IgAV adult patients with four HCs. They found significantly elevated serum IgA and MPO-DNA levels that positively correlated in patients compared with HC, suggesting NETs may be associated with IgAV disease activity. They reported higher IgA, MPO-DNA levels, and proteinuria frequency in ANCA-positive patients. Conversely, our study found lower serum MPO in paediatric patients with no relation to renal involvement [23]. This contradiction may reflect differences between paediatric and adult IgAV mechanisms. Methodological differences may also contribute, as Takeuchi’s study had a smaller sample size and focused on ANCA relationships, while ours used a larger control group and examined multiple NETosis markers simultaneously.

Chen *et al.* compared 193 paediatric IgAV patients with 192 HCs at different stages of IgAV. Serum cf-DNA, MPO, NE and DNase I levels and blood NET degradation were analysed simultaneously with kidney biopsies in 15 patients and gastrointestinal biopsies in 9 patients. NETs markers in serum were found to be elevated during the active period, while serum DNase I levels and NET degradation were diminished. The presence of NET was confirmed in tissue biopsy samples [15]. The authors suggested that NETosis markers could serve as potential disease activity indicators. Our study found elevated cf-DNA levels in active and inactive IgAV phases, while other NETosis markers showed no significant serum increase. The discrepancy with Chen *et al.* may stem from differences in patient characteristics or sampling timing. This highlights IgAV pathogenesis heterogeneity, suggesting that the role of NETs may vary depending on genetic backgrounds, environmental triggers or disease subtypes. It is emphasized that standard approaches are needed to reveal the clinical significance of these markers in future research.

Within the scope of the role of NETosis in vasculitis, Yoshida *et al.* studied NETs in Kawasaki disease, evaluating 37 patients in active and convalescent phases against 6 controls [24]. Using IF, PCR and ELISA methods, they found NETosis markers were elevated during the active phase, consistent with our findings. Disease follow-up included microscopic examination in 11 patients, while serum cf-DNA and NE levels were evaluated by PCR and ELISA in 15 patients during active and convalescent periods, showing elevation in the active phase. Despite differences between these vasculitis types, the consistent elevation of cf-DNA in the active phase points to the potential role of NETosis as a fundamental mechanism in endothelial damage across vasculitis subtypes. Therapeutic approaches targeting NET formation may have applications across various vasculitic disorders, especially during active inflammation when vascular damage occurs.

MPO, essential for neutrophil microbial elimination, shows controversial findings across studies. Our research demonstrated significantly elevated serum and urine MPO levels in controls, contradicting Chen *et al.*’s findings of increased levels in IgAV patients [15]. While some adult studies reported elevated MPO levels [25–27], a BD study showed higher MPO levels in controls [25]. This study revealed decreased activities of MPO, superoxide dismutase, catalase and glutathione peroxidase in BD patients, potentially due to PMN granule depletion from systemic activation. This mechanism, coupled with infection-triggered IgAV, potentially compromises MPO-mediated microbicidal function, causing endothelial injury through increased free oxygen exposure. The low serum MPO levels in our study differ from previous reports, possibly due to population differences, methodology or sampling timing. The exact mechanism requires further tissue-level investigation [15, 23].

Histone citrullination by peptidyl arginine deiminases is a key posttranslational modification enabling chromatin decondensation during NET formation. Citrullinated histones, particularly Cit-H3, are found in the extracellular space as NET components and are highly involved in NETosis. In our study, serum cit-H3 levels showed no difference between groups, but urine levels were significantly higher in both active and inactive phases compared with controls. A study in Kawasaki disease found elevated serum cit-H3 along with other NETosis markers compared with healthy subjects [28]. They speculated that cit-H3 may damage vascular endothelial cells, increasing vascular permeability and causing further damage to small- and medium-sized blood vessels in Kawasaki disease. A study on vasculitis with renal involvement found tissue accumulation of high cit-H3 levels associated with disease activity [29]. Similarly, Bergqvist *et al.* found elevated cit-H3 levels in small vessel vasculitis (SVV) skin biopsies. However, these two studies did not analyse serum and urine samples [22]. Parallel to these studies, in a report providing evidence on the implication of NETosis in the pathophysiology of BD, cit-H3 was invariably present in all NETs from BD neutrophils [13]. Considering our study, high urine cit-H3 with low serum levels may reflect complex marker distribution and clearance dynamics, though tissue-level studies are needed to fully understand the mechanisms.

When the findings of this study and literature data are considered holistically, the clinical significance of cf-DNA and urinary cit-H3 levels is demonstrated by several key findings. Serum cf-DNA levels showed significant elevation during active disease compared with controls (*P* = 0.04), with high sensitivity (93%) and specificity (72%). Its normalization during inactive phases may suggest its potential utility as a disease activity biomarker. Urinary cit-H3 levels showed significant elevation in both active and inactive phases compared with controls (*P* = 0.01 and *P* = 0.03, respectively), demonstrating high sensitivity (85%) for active disease detection. The positive correlation between serum and urinary cit-H3 levels during the active phase, coupled with their persistent elevation, may suggest their potential role in monitoring disease activity and progression. Both markers may be candidate tools for disease monitoring and therapeutic decision-making in IgAV management.

One of the superiorities of our study is determining the cut-off value of serum cf-DNA (935 ng/ml) for identifying active patients through ROC curve analysis (93% sensitivity and 72% specificity). In a recent study, ROC curve analysis of cf-DNA was performed in juvenile SpA patients and the cut-off values were consistent with our study [30]. Therefore, cf-DNA may be a potential biomarker to distinguish active from inactive patients.

This study has limitations including a relatively small patient number for disease severity grouping. The analysis relied on a single method for the measurement of NET levels, however this may prevent the generation of inconclusive results. The inability to assess the relationship between D-dimer, factor XIII and NETosis markers is the other limitation of our study and represents a planned direction for our future research.

In conclusion, conducted longitudinally with a HC group, this study presents the first analysis of urinary NETosis markers in paediatric IgAV patients, potentially offering a noninvasive alternative to biopsy for disease monitoring. We establish a specific serum cf-DNA cut-off value (935 ng/ml) with high sensitivity for detecting active disease. While NETosis markers have been extensively studied in adults, paediatric research remains limited. These findings enhance our understanding of IgAV pathogenesis and introduce practical, noninvasive monitoring tools that could improve paediatric patient care by providing measurable disease activity parameters, addressing gaps in paediatric vasculitis management. Further research in larger cohorts is needed to verify these findings and better understand the implications.

## Data Availability

The data underlying this article will be shared on reasonable request to the corresponding author.
